# Health Effects of Coffee: Mechanism Unraveled?

**DOI:** 10.3390/nu12061842

**Published:** 2020-06-20

**Authors:** Hubert Kolb, Kerstin Kempf, Stephan Martin

**Affiliations:** 1 Faculty of Medicine, University of Duesseldorf, Moorenstr. 5, 40225 Duesseldorf, Germany; hubert.kolb@hhu.de (H.K.); stephan.martin@uni-duesseldorf.de (S.M.); 2 West-German Centre of Diabetes and Health, Duesseldorf Catholic Hospital Group, Hohensandweg 37, 40591 Duesseldorf, Germany

**Keywords:** coffee, phytochemicals, caffeine, diabetes, DNA damage, antioxidant, Nrf2, microbiota

## Abstract

The association of habitual coffee consumption with a lower risk of diseases, like type 2 diabetes mellitus, chronic liver disease, certain cancer types, or with reduced all-cause mortality, has been confirmed in prospective cohort studies in many regions of the world. The molecular mechanism is still unresolved. The radical-scavenging and anti-inflammatory activity of coffee constituents is too weak to account for such effects. We argue here that coffee as a plant food has similar beneficial properties to many vegetables and fruits. Recent studies have identified a health promoting mechanism common to coffee, vegetables and fruits, i.e., the activation of an adaptive cellular response characterized by the upregulation of proteins involved in cell protection, notably antioxidant, detoxifying and repair enzymes. Key to this response is the activation of the Nrf2 (Nuclear factor erythroid 2-related factor-2) system by phenolic phytochemicals, which induces the expression of cell defense genes. Coffee plays a dominant role in that regard because it is the major dietary source of phenolic acids and polyphenols in the developed world. A possible supportive action may be the modulation of the gut microbiota by non-digested prebiotic constituents of coffee, but the available data are still scarce. We conclude that coffee employs similar pathways of promoting health as assumed for other vegetables and fruits. Coffee beans may be viewed as healthy vegetable food and a main supplier of dietary phenolic phytochemicals.

## 1. Introduction

In recent years, numerous meta-analyses have come up with positive health outcomes associated with habitual coffee consumption in the general population, and this has changed the perception of coffee from that of a luxury stimulant drink to that of a health promoting beverage, if consumed within usual levels of intake. Positive health outcomes include lower incidences of type 2 diabetes mellitus, kidney stones, Parkinson’s disease, gout, liver fibrosis, non-alcoholic fatty liver disease, liver cirrhosis, liver cancer and of chronic liver disease. This is the conclusion of an umbrella review of meta-analyses of multiple health outcomes, even after extensive correction for a large number of possible confounding factors [1], and also the result of the EPIC (European Prospective Investigation into Cancer and Nutrition Study) trial analyzing coffee consumption versus mortality [2]. Consumption of decaffeinated coffee was associated with similar beneficial outcomes, but only if data of large cohorts were available [3,4,5]. The molecular mechanism responsible for these putative health effects is still unresolved.

Epidemiological studies cannot prove causality, but it is remarkable that assumed health effects such as a lower risk of type 2 diabetes are seen at a global level, in different regions with different cultures and lifestyle. Moreover, a dose-response relationship between number of cups of coffee consumed per day and diabetes risk was observed, which is difficult to explain by an overlooked lifestyle factor [6].

However, it should not be ignored that heavy consumption of coffee may have a genetic basis, and that the latter accounts for better health outcomes. Drinking several cups of coffee per day would only be a marker of a favorable genetic background. Genome-wide association studies have identified an impact of several gene polymorphisms on caffeine or coffee intake [7,8,9]. Caffeine seems to be relevant for most genes identified, notably the gene CYP1A2 (cytochrome P450 isozyme 1A2) which is involved in the hepatic metabolism of caffeine. Carriers of the C variant at position 163 express less CYP1A2 and therefore metabolize caffeine more slowly than persons homozygous for the A allele [10]. Faster breakdown of caffeine is associated with more caffeine or coffee consumption.

Mendelian randomization studies made use of the finding that some genotypes are associated with increased coffee consumption. Nonetheless, there were no consistent associations of the genotype for faster caffeine metabolism (and more caffeine/coffee consumption) with positive health outcomes [11]. In spite of this, these findings do not invalidate the association of coffee consumption with health effects for several reasons. The difference between the high and low caffeine consumption genotypes of CYP1A2 is about 40 mg of caffeine, i.e., less than half a cup of coffee [7]. Habitual coffee consumption ranges from about 1 to more than 5 cups per day, which indicates that the daily dose is defined by something other than genetic reasons. Epidemiological studies find significant associations for cohorts that differ by consumption of 2 or more cups of coffee [1]. Among persons with the same CYP1A2 genotype, those drinking more coffee show better health outcomes, such as concerning Parkinson’s disease or breast cancer [12,13]. Changes in coffee consumption were accompanied by a parallel change of health risk, i.e., type 2 diabetes. This argues against a major influence of genetic characteristics as well [14].

An epidemiological study of nearly 500,000 participants of the United Kingdom Biobank finds associations between the number of cups of coffee consumed per day and decreased all-cause mortality, regardless of genetic caffeine metabolism score, i.e., the circadian level of caffeine in circulation [15]. Caffeine also does not appear to account for the lower risk of type 2 diabetes with habitual coffee consumption, since this is also seen in association with drinking decaffeinated coffee. Impairment of glucose tolerance is observed after consumption of caffeinated, but not decaffeinated coffee, suggesting that other phytochemicals in coffee outweigh possible detrimental effects of caffeine. It has been suggested that polyphenols and other bioactives in decaffeinated coffee mediate these health effects [16].

**Figure 1:**
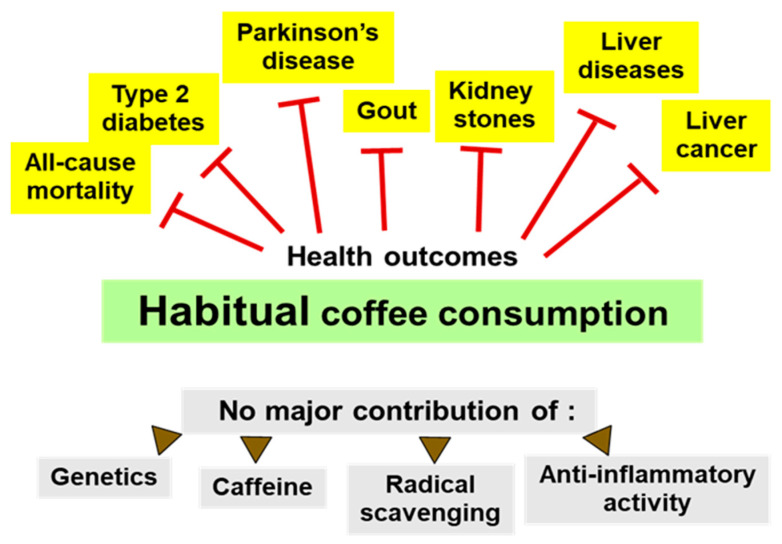
Health outcomes of coffee consumption. Prospective epidemiological studies consistently found a lower risk of several clinical outcomes and of all-cause mortality for habitual coffee consumption [1,2]. Most of these associations cannot be accounted for by genetic polymorphisms promoting coffee/caffeine consumption, by the caffeine content of coffee, or by its content of radical scavenging or anti-inflammatory constituents. Health effects of decaffeinated coffee could only be determined if sufficiently large cohorts were available for study. Otherwise, positive trends did not reach statistical significance, such as for Parkinson’s disease.

We conclude that the possible mechanism of coffee-mediated health effects does not include a major role of caffeine actions (Figure 1). An important health promoting role of vitamins and minerals in the coffee brew seems improbable because, on average, there is no deficient intake in the developed world. Health effects therefore appear to be associated with other prominent constituents of the coffee brew. These include chlorogenic acids, trigonelline, N-methylpyridinium, the diterpenes kahweol and cafestol, polysaccharides, peptides and melanoidins. For several of these components, radical scavenging or anti-inflammatory activity has been postulated. We argue here that such hypotheses do not fit with the available data. Rather, beneficial effects of coffee probably employ the same pathway as recently suggested for “healthy” vegetables or fruits, i.e., the induction of a health promoting adaptive response of cells in the body. Additionally, non-digestible components of coffee may modulate the composition and function of the microbiota, as is known for other plant foods.

## 2. Inefficient Radical Scavenging by Coffee Constituents

Several phenolic components of the coffee brew exhibit radical scavenging properties, which increase in quantity during roasting [17,18]. Although radical scavenging by ingested coffee components is still a quite common belief, there is ample evidence from animal and human studies showing that the concentrations of coffee constituents reached in plasma are too low for efficient radical scavenging. After consumption of coffee, peak plasma concentrations of phenolic metabolites range between 0.01 and 6 µmol/L, and caffeine concentrations may reach 30 µmol/L [19]. These values compare well with peak flavonoid concentrations seen in human plasma after consumption of 100 g of fruits and vegetables, which range between 0.03 µmol/L for apples and 5.9 µmol/L for cocoa [20]. Such concentrations are well below levels of endogenous antioxidant systems such as urate (160–450 µmol/L), ascorbate (30–150 µmol/L), α-tocopherol (15–40 µmol/L), glutamine (~500 µmol/L) or glutathione (>1 mmol/L in cells) [20,21,22]. In addition, the major coffee constituents caffeoylquinic acids, trigonelline and caffeine are weak antioxidants (one-electron reduction potential) in comparison to vitamins C or E, or glutathione (reviewed in Reference [23]), and thus cannot effectively reduce/regenerate oxidized forms of antioxidant vitamins or glutathione. Thus, radical scavenging by coffee components in vivo is limited and probably contributes little to the health effects of coffee (Figure 1). As discussed below, there is an antioxidative effect of coffee consumption because of the induction of endogenous radical scavenging enzymes.

## 3. Weak Anti-Inflammatory Action of Coffee

Cross-sectional studies of the level of circulating immune or inflammatory markers have reported small and not consistent variations in relation to habitual coffee consumption [24,25]. Randomized controlled trials of several weeks of coffee consumption in comparison to a water control were also performed and small reductions of some immune/inflammatory mediator concentrations were found, but the opposite was also reported [26,27,28,29,30]. Medium, but not dark roast coffee consumption increased the level of adiponectin [31]. A modulatory effect of habitual coffee consumption on the risk of an inflammatory disease, rheumatoid arthritis, was not observed [1,32]. Taken together, there may be a mild favorable anti-inflammatory response of the immune system to coffee consumption, but possible effects do not appear to reach clinical significance (Figure 1).

## 4. Phenolic Phytochemicals in Coffee May Account for Health Effects

Besides caffeine, major constituents of coffee are of a phenolic nature. These include roasting-induced degradation products of chlorogenic acids (caffeoylquinic acids), trigonelline and its roasting product, N-methylpyridinium. Melanoidins are also major components, these are roasting-dependent Maillard reaction products of carbohydrate residues with amino acids or protein side chains.

Coffee is entirely of plant origin. Thus, it is conceivable that its consumption causes similar health promoting responses in the human organism as described for many other plant foods. Virtually all plant-derived foods contain health promoting phytochemicals, and most of them are of a phenolic nature [33,34]. At present, there is no reason to assume that phenolic compounds of coffee are less “healthy” than comparable phytochemicals of tea, vegetables or fruits. However, coffee sticks out in one important regard: in habitual coffee drinkers, coffee is the primary dietary source of phytochemicals like phenolic acids and polyphenols, even in comparison to green tea in Japan [35,36,37,38,39]. At the level of populations, coffee provides around 40% of polyphenols and around 70% of phenolic acids consumed, followed by tea as the second major source.

**Figure 2:**
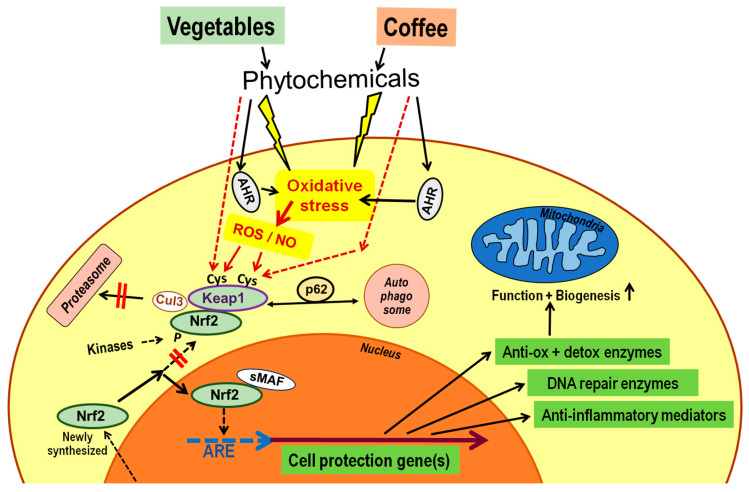
Phytochemicals activate the nuclear factor erythroid 2-related factor 2 (Nrf2) pathway. Exposure of cells to phenolics of vegetables or coffee leads to cell stress, including oxidative stress via mostly unknown pathways, except for some involvement of the aryl hydrocarbon receptor (AHR). Major sources of radical oxygen species (ROS) during oxidative stress are the mitochondrial respiratory chain and nicotinamide adenine dinucleotide phosphate (NADPH) oxidases of the NOX family. The nuclear factor Nrf2 is usually bound to Keap1 and the Cullin 3-based E3 ubiquitin ligase (Cul3), which is followed by transport to the proteasome for degradation. This process can be blocked by modification of one or more cysteine residues of Kelch-like ECH-associated protein 1 (Keap1) by ROS, ROS-modified fatty acids or cyclic nucleotides, by nitric oxide (NO), or by direct action of phytochemical electrophiles. The protein p62 blocks binding of Nrf2 to Keap1 and channels Keap1 to autophagic destruction. Several kinases, such as members of the Src family, can phosphorylate Nrf2 and may also interfere with routing to proteasomes. All these mechanisms prevent newly formed Nrf2 from being captured by Keap1, so that translocation to the nucleus is possible. In the nucleus, Nrf2 binds to small musculoaponeurotic fibrosarcoma (sMaf) protein, and the heterodimer interacts with the antioxidant response element (ARE) upstream of several hundred genes involved in cell defense mechanisms, resulting in enhanced transcription. The following points are not depicted in the scheme: AHR forms a complex with several other proteins including heat shock protein (hsp) 90. AHR activation by selective phytochemicals not only increases intracellular oxidative stress but also leads to translocation of the factor to the nucleus where it upregulates a set of genes involved in xenobiotic defense and immunoregulation. Some of the intranuclear Nrf2 molecules are phosphorylated. Nrf2 gene expression can be modified by affecting its transcription, such as by inhibitory microRNAs; additionally, Keap1 gene expression can also be modified.

We therefore propose that coffee employs similar molecular pathways for improving health as described for other plant foods such as broccoli, beetroot, berries, pomegranate, curcuma, cocoa and many others. Surprisingly, there seems to be one uniform response of cells when exposed to phenolic phytochemicals or their metabolites at concentrations observed in vivo after a meal, despite major differences in chemical structure of phenolic compounds. The cellular response is characterized by an increased expression of a large number of genes involved in antioxidative, detoxifying or repair mechanisms, this is also observed in vivo [40]. The molecular pathway involves the translocation of nuclear factor erythroid 2–related factor 2 (Nrf2) from cytosol to the nucleus, formation of heterodimers with small musculoaponeurotic fibrosarcoma (sMaf) proteins and binding to consensus DNA sequences, referred to as antioxidant response elements (ARE), electrophile response elements and more recently as cap’n’collar (CNC)-sMaf binding elements [41]. The response elements are present in the 5′-upstream region of several hundred cytoprotective genes, and binding of Nrf2/sMaf gives rise to increased gene expression of proteins involved in cell defense. These include antioxidant enzymes such as superoxide dismutase, catalase, glutathione peroxidase, glutamate-cysteine ligase and xenobiotic detoxifying enzymes, including nicotinamide adenine dinucleotide phosphate (NAD(P)H):quinone oxidoreductase-1, uridine 5′-diphospho (UDP)-glucuronosyltransferases or heme oxygenase-1 [42]. Decreased gene expression is seen for pro-inflammatory mediators like tumor necrosis factor-α or the NLRP3 (NOD-like receptor family, pyrin domain containing 3) inflammasome. Activation of Nrf2 is also required for the induction of mitochondrial biogenesis and antioxidant response. Furthermore, there is a regulation of substrate supply to mitochondria by Nrf2 [43,44,45] (Figure 2).

Under steady physiological conditions, the majority of newly synthesized Nrf2 is captured by the repressor protein Kelch-like ECH-associated protein 1 (Keap1) and channeled to proteasomal degradation via ubiquitylation by Cullin 3-based E3 ubiquitin ligase (Cul3). Keap1 is the main cellular sensor for stress molecules due to expression of 17 cysteine residues which are targets for modification by radical oxygen species (ROS), ROS-modified fatty acids or cyclic nucleotides, nitric oxide (NO) or other electrophiles [46]. Any modification suppresses the ability of Keap1 to transfer Nrf2 to proteasomes, which prevents Keap1 from capturing new Nrf2 molecules so that newly synthesized Nrf2 can translocate to the nucleus [47]. Phytochemicals may either directly target cysteines of Keap1, such as sulforaphane, or modify cell functions, resulting in oxidative stress and subsequent inactivation of Keap1. The interaction of different electrophiles with Keap1 leads to different patterns of modified cysteine residues, and to the activation of different patterns of cytoprotective genes, which may in part explain the different responses of cells to different phytochemicals [46].

Enhancement of Nrf2 activity may also result from blocking its binding to Keap1 by the autophagy adaptor protein p62, which channels Keap1 to degradation in autophagosomes. Proteasomal destruction of Nrf2 can also be inhibited via phosphorylation by several protein kinases, such as members of the Src family, by induction of transcription factors for the increased expression of Nrf2, or the downregulation of inhibitory microRNAs or of Keap1 expression [40,47,48] (Figure 2).

In order to prove that the adaptive response to plant phytochemicals indeed requires activation of the Nrf2 pathway, studies were performed with cells or animals with an inactivated or deleted Nrf2 gene. In the absence of Nrf2, all phytochemicals studied lost their cell protective activity. Phytochemicals included quercetin, epigallocatechin gallate (EGCG), resveratrol, isothiocyanates, allicin, curcumin and aspalathin [40,49,50,51,52]. The adaptive response to dietary phytochemicals often includes some anti-inflammatory activity, via suppression of nuclear factor kB (NFkB), which is the master regulator of inflammatory reactivity. This effect is either Nrf2-dependent or may be due to direct targeting of transcription factors NFkB or activator protein 1 by phytochemicals [40].

A second chemical sensor, engaged by phytochemicals as ligands, is the aryl hydrocarbon receptor (AHR), a transcription factor in the cytoplasm promoting the production of radical oxygen species and counterregulatory Nrf2 activity (Figure 2). Upon ligand binding, AHR is released from the complex with heat shock protein (hsp) 90 and is transported to the nucleus, where it dimerizes with the aryl hydrocarbon receptor nuclear translocator, followed by binding to AHR response elements upstream of a set of genes coding for detoxifying enzymes, such as the cytochrome P450 family 1 and immunoregulatory mediators [53,54,55] (not depicted in Figure 2).

## 5. Phenolic Constituents of Coffee Activate the Nrf2 Pathway

Exposure of cells or animals to coffee extracts has been observed to lead to increased expression of cytoprotective genes involved in the antioxidant defense, as well as in other chemoprotective or repair activities [56,57,58,59,60,61,62]. Decreased gene expression is seen for pro-inflammatory mediators like tumor necrosis factor-α or the NLRP3 inflammasome [60,63].

As seen for phytochemicals of other plants, these effects are mediated by the activation of the Nrf2 system and by inhibiting the pro-inflammatory NFkB pathway (Figure 2). The cytoprotective response seen in mice after consumption of coffee was suppressed after inactivation of the Nrf2 gene [64]. Many constituents of coffee can activate the Nrf2 pathway, with melanoidins of dark roast coffee contributing to these effects [56,57,58,60,61,62,65,66,67,68]. The diterpenes cafestol and kahweol present in coffee are known to elevate plasma low-density lipoprotein (LDL) and triacylglycerol concentration, while also exhibiting anti-inflammatory and antioxidant actions [69]. The latter cytoprotective action is also mediated by the Nrf2 system and is absent in Nrf2 gene knockout mice [64]. Several of the animal studies took care to apply doses of coffee extract comparable to the consumption of 2–5 cups of coffee in human adults [57,59,60,61].

Roasting of green coffee increases the ability to activate the Nrf2 pathway. In addition to this, dark roast coffee is more potent in that regard than light roast coffee, when analyzed in vivo [68,70,71]. The analysis of single coffee constituents has confirmed the differing activity of light versus dark roast coffee. Whereas N-methylpyridinium appears to be an activator of Nrf2 as potent as caffeoylquinic acids [56,66], trigonelline suppressed the activation of Nrf2. The lower content of trigonelline fits with the stronger activation of Nrf2 by dark roast coffee [56]. These effects in vitro were observed at physiologically relevant concentrations of 0.1 µmol/L [56]. Moreover, the studies of other constituents of coffee, such as chlorogenic acids, caffeic acid or kahweol, observed activation of Nrf2 at concentrations varying between 10 nmol/L and 3 µmol/L [56,62,65,66,67], which are in the range of peak concentrations in human plasma after coffee consumption.

Taken together, a large number of trials has shown that coffee or several of its isolated constituents are potent activators of antioxidant or cell-protective enzyme expression. Dark roast coffee appears to be more potent in that regard than light roast coffee. The available evidence indicates that the activation of Nrf2 pathways (to some extent via the aryl hydrocarbon receptor) is the major pathway involved.

Phenolic compounds are characterized in part by hydrophobic surface areas of the molecule. These regions tend to bind to hydrophobic pockets of accessible proteins in tissues and may cause their denaturation and aggregation, which can lead to cell stress, including oxidative stress, and activation of the Nrf2 system as a protective response. High doses of phenolic compounds can be cytotoxic [72,73,74], but these conditions are usually not reached after a meal of plant foods. For instance, an upper safe limit for the ingestion of green tea catechins has been defined and set as 800 mg epigallocatechin-3-gallate [75]. The phenomenon that tolerable doses of potential toxins induce increased resistance to the same or other chemical insults has been initially observed in toxicological research and defined as hormesis [76] and has been extended to the action of polyphenols [77]. Hormetic reaction schemes have been found or suggested to underlie many physiological processes [78,79,80].

Although the induction of a cytoprotective response in cells appears to be the dominant physiological reaction to coffee consumption, evidence for a cause–effect relationship with health outcomes is lacking, except for the beneficial effect of coffee intake on DNA integrity. Several randomized-controlled trials have observed that after a run-in period and 4–8 weeks of coffee or water consumption, values of spontaneous DNA strand breaks in blood lymphocytes were significantly lower in the coffee group, as determined by comet assay [81,82,83]. In one center, the initial trial [81] was repeated with a similar study protocol but the difference in favor of the coffee group was too small to be significant [84]. The level of spontaneous DNA strand breaks in blood lymphocytes is a relevant marker of general disease risk evaluated in epidemiological studies. A meta-analysis of 122 studies described significantly less disease risks for an 11–58% lower level of spontaneous DNA strand breaks, as determined by comet assay [85]. Coffee consumption (of a dark roast Arabica coffee blend shown to activate Nrf2), in three trials described above, resulted in 16–35% less spontaneous DNA strand breaks. These data indicate a cause–effect relationship between coffee consumption and a lower level of spontaneous DNA strand breaks, and that the effect is of physiologically relevant magnitude.

## 6. Other Possible Pathways of Coffee-Mediated Health Effects

Additional health promoting effects of coffee may occur in the gut, not requiring uptake and biochemical modification of coffee components. Indeed, a prebiotic effect of coffee consumption has been observed in animals and humans, including an increase of Bifidobacteria in humans and mice, and modulation of the Firmicutes to Bacteroidetes ratio in rats. In mice, feeding coffee led to higher levels of acetate, propionate and butyrate [86,87,88]. Candidate prebiotic constituents of coffee are soluble arabinogalactans and galactomannans, melanoidins and polyphenols [89,90,91]. However, in male Tsumura Suzuki obese diabetes mice, a mouse model of metabolic syndrome, daily coffee intake prevented nonalcoholic steatohepatitis but did not repair the altered levels of Gram-positive and Gram-negative bacteria and the increased abundance of Firmicutes, nor was there improvement of the disrupted short chain fatty acid profile [92]. Interestingly, feeding probiotic *Lactobacilli* caused the upregulation of Nrf2 in the liver, with concomitant resistance to oxidative injury [93].

To mediate long-term health effects, changes of the microbiota induced by coffee consumption needed to last for decades. A recent study observed that the initial substantial changes of the microbiota, seen after 3 months of introducing a specific diet, regressed thereafter to the original baseline state, which persisted despite continuation of the experimental diet for 12 months [94]. Similar trials of long-term coffee consumption have not been performed, thus the role of the gut microbiota in mediating health effects of coffee constituents remains unresolved.

## 7. Phytochemicals and Health—A Broader Perspective

One major function of phytochemicals, including those of *coffea* species, is to confer protection from environmental challenges such as exposure to UV radiation or toxins, and to prevent being eaten or damaged by pests or insects because of their noxious properties. At the relatively small doses ingested and taken up by humans, edible parts of plants are not toxic but induce a biological stress response [95]. Besides activation of the Nrf2 system, a number of other cellular responses have been noted, such as the stimulation of the sirtuin-forkhead box O pathway, of AMP-activated protein kinase, of mitogen-activated protein kinases, of the phosphatidylinositol-3-kinase (PI3K)—serine/threonine-specific protein kinase B (AKT) pathway, or modulation of the nuclear factor kB pathway [95,96,97]. Many outcomes of stress signaling are beneficial, such as the stimulation of antioxidant activity, of anti-inflammatory activity, of mitochondrial function, of DNA repair, of autophagy, of various metabolic parameters and of cell rejuvenation or apoptosis [98].

The many different signaling pathways involved in the cellular response to stress are interdependent and are part of a regulated network. Several studies have suggested a central role for the Nrf2 system. For example, in the absence of Nrf2 gene activity, the mitochondria-dependent protection by broccoli extract or sulforaphane from pulmonary injury is almost abolished [49], as is the PI3K-AKT-dependent protection by Withania from liver injury [99], or the hemooxygenase-1-dependent protection by curcumin or phenethyl isothiocyanate from inflammatory stress [50]. The extracellular-signal-regulated kinase (ERK) forms a signaling pathway together with Nrf2 [100,101], as does AMPK with Nrf2 [102]. Sirtuin-1 and Nrf2 represent another joint pathway induced by phytochemicals [103].

It thus appears justified to consider the Nrf2 system as a central regulator of phytochemical-induced stress defenses. The type of cellular response may depend on chemical properties of the phytochemical studied, the cell type and developmental stage and further physiological factors.

## 8. Conclusions

Phytochemicals other than caffeine appear to account for most beneficial properties of coffee. As in vegetables or fruits, polyphenols and phenolic acids represent a major portion of phytochemicals in coffee beans. Phenolic phytochemicals of plant foods have one major pathway of health promoting effects in common, the induction of cell stress, which leads to the activation of an adaptive cell defense response, via activation of the Nrf2 system, translocation of Nrf2 to the nucleus and increased expression of Nrf2-dependent antioxidant and other cytoprotective genes. Furthermore, there is an improved biogenesis, antioxidant defense and substrate supply to mitochondria. Activation of the Nrf2 system and the subsequent cell defense response is also observed in response to exposure with coffee. Randomized controlled trials have found an improved preservation of DNA integrity after several weeks of coffee consumption, this outcome is also assumed to be mediated by the Nrf2 system. Polyphenols and other nondigestible constituents of coffee like polysaccharides and melanoidins share the ability to modify the composition and metabolic function of gut microbiota with similar components of other plant foods. Whether these effects contribute to the beneficial properties of coffee remains to be studied. Taken together, coffee employs similar pathways of beneficial physiological effects as were recently identified for vegetables or fruits. At the level of populations in the developed world, coffee provides more dietary phenolic phytochemicals than vegetables and fruits (Box 1).

Box 1 **Key messages.**

1. Habitual coffee consumption is associated with a lower risk of many chronic diseases and all-cause mortality.
2. The contribution of oxygen radical scavenging by coffee constituents to health effects is apparently small.
3. Coffee is a plant food and the majority of dietary phenolics consumed in the developed world come from coffee.
4. It is suggested that phenolic constituents of coffee exhibit similar health promoting effects as those from vegetables or fruits.
5. The main pathway of health effects of phenolic phytochemicals from plant food, as well as from coffee, is the activation of the Nrf2 system for an adaptive cytoprotective response.
6. Nrf2-dependent genes code for proteins with antioxidative, detoxifying, DNA repair or anti-inflammatory functions.

## Abbreviations

AHR	aryl hydrocarbon receptor
ARE	antioxidant response elements
CNC	cap’n’collar
Cul3	Cullin 3-based E3 ubiquitin ligase
CYP1A2	cytochrome P450 isozyme 1A2
DNA	desoxyribonucleic acid
EGCG	epigallocatechin gallate
EPIC	European Prospective Investigation into Cancer and Nutrition Study
HSP	heat shock protein
IRS	insulin receptor substrate
Keap1	Kelch-like ECH-associated protein 1
LDL	Low-density lipoprotein
NADPH	nicotinamide adenine dinucleotide phosphate
NFkB	nuclear factor kB
NLRP3	NOD-like receptor family, pyrin domain containing 3
NO	nitric oxide
Nrf2	Nuclear factor erythroid 2-related factor 2
ROS	radical oxygen species
sMaf	small musculoaponeurotic fibrosarcoma
UDP	uridine 5′-diphosphate

## References

[1] Poole R., Kennedy O.J., Roderick P., Fallowfield J.A., Hayes P.C., Parkes J. (2017). Coffee consumption and health: Umbrella review of meta-analyses of multiple health outcomes. BMJ.

[2] Gunter M.J., Murphy N., Cross A.J., Dossus L., Dartois L., Fagherazzi G., Kaaks R., Kuhn T., Boeing H., Aleksandrova K. (2017). Coffee drinking and mortality in 10 European countries: A multinational cohort study. Ann. Intern. Med..

[3] Carlstrom M., Larsson S.C. (2018). Coffee consumption and reduced risk of developing type 2 diabetes: A systematic review with meta-analysis. Nutr. Rev..

[4] Je Y., Giovannucci E. (2014). Coffee consumption and total mortality: A meta-analysis of twenty prospective cohort studies. Br. J. Nutr..

[5] Li Q., Liu Y., Sun X., Yin Z., Li H., Cheng C., Liu L., Zhang R., Liu F., Zhou Q. (2019). Caffeinated and decaffeinated coffee consumption and risk of all-cause mortality: A dose-response meta-analysis of cohort studies. J. Hum. Nutr. Diet.

[6] Cornelis M.C. (2020). Coffee and type 2 diabetes: Time to consider alternative mechanisms?. Am. J. Clin. Nutr..

[7] Cornelis M.C., Monda K.L., Yu K., Paynter N., Azzato E.M., Bennett S.N., Berndt S.I., Boerwinkle E., Chanock S., Chatterjee N. (2011). Genome-wide meta-analysis identifies regions on 7p21 (AHR) and 15q24 (CYP1A2) as determinants of habitual caffeine consumption. PLoS Genet..

[8] Amin N., Byrne E., Johnson J., Chenevix-Trench G., Walter S., Nolte I.M., Vink J.M., Rawal R., Mangino M., Teumer A. (2012). Genome-wide association analysis of coffee drinking suggests association with CYP1A1/CYP1A2 and NRCAM. Mol. Psychiatry.

[9] Cornelis M.C., Byrne E.M., Esko T., Nalls M.A., Ganna A., Paynter N., Monda K.L., Amin N., Fischer K., Renstrom F. (2015). Genome-wide meta-analysis identifies six novel loci associated with habitual coffee consumption. Mol. Psychiatry.

[10] Nehlig A. (2018). Interindividual differences in caffeine metabolism and factors driving caffeine consumption. Pharmacol. Rev..

[11] Cornelis M.C., Munafo M.R. (2018). Mendelian randomization studies of coffee and caffeine consumption. Nutrients.

[12] Popat R.A., Van Den Eeden S.K., Tanner C.M., Kamel F., Umbach D.M., Marder K., Mayeux R., Ritz B., Ross G.W., Petrovitch H. (2011). Coffee, ADORA2A, and CYP1A2: The caffeine connection in Parkinson’s disease. Eur. J. Neurol..

[13] Kotsopoulos J., Ghadirian P., El Sohemy A., Lynch H.T., Snyder C., Daly M., Domchek S., Randall S., Karlan B., Zhang P. (2007). The CYP1A2 genotype modifies the association between coffee consumption and breast cancer risk among BRCA1 mutation carriers. Cancer Epidemiol. Biomarkers Prev..

[14] Bhupathiraju S.N., Pan A., Manson J.E., Willett W.C., Van Dam R.M., Hu F.B. (2014). Changes in coffee intake and subsequent risk of type 2 diabetes: Three large cohorts of US men and women. Diabetologia.

[15] Loftfield E., Cornelis M.C., Caporaso N., Yu K., Sinha R., Freedman N. (2018). Association of coffee drinking with mortality by genetic variation in caffeine metabolism: Findings from the UK Biobank. JAMA Intern. Med..

[16] Palatini P. (2015). Coffee consumption and risk of type 2 diabetes. Diabetologia.

[17] Opitz S.E., Goodman B.A., Keller M., Smrke S., Wellinger M., Schenker S., Yeretzian C. (2017). Understanding the effects of roasting on antioxidant components of coffee brews by coupling on-line ABTS assay to high performance size exclusion chromatography. Phytochem. Anal..

[18] Kamiyama M., Moon J.K., Jang H.W., Shibamoto T. (2015). Role of degradation products of chlorogenic acid in the antioxidant activity of roasted coffee. J. Agric. Food Chem..

[19] Lang R., Dieminger N., Beusch A., Lee Y.M., Dunkel A., Suess B., Skurk T., Wahl A., Hauner H., Hofmann T. (2013). Bioappearance and pharmacokinetics of bioactives upon coffee consumption. Anal. Bioanal. Chem..

[20] Lotito S.B., Frei B. (2006). Consumption of flavonoid-rich foods and increased plasma antioxidant capacity in humans: Cause, consequence, or epiphenomenon?. Free Radic. Biol. Med..

[21] Lee Y.H. (2019). Coffee consumption and gout: A Mendelian randomisation study. Ann. Rheum. Dis..

[22] Giustarini D., Colombo G., Garavaglia M.L., Astori E., Portinaro N.M., Reggiani F., Badalamenti S., Aloisi A.M., Santucci A., Rossi R. (2017). Assessment of glutathione/glutathione disulphide ratio and S-glutathionylated proteins in human blood, solid tissues, and cultured cells. Free Radic. Biol. Med..

[23] Ludwig I.A., Clifford M.N., Lean M.E., Ashihara H., Crozier A. (2014). Coffee: Biochemistry and potential impact on health. Food Funct..

[24] Calder P.C., Ahluwalia N., Brouns F., Buetler T., Clement K., Cunningham K., Esposito K., Jonsson L.S., Kolb H., Lansink M. (2011). Dietary factors and low-grade inflammation in relation to overweight and obesity. Br. J. Nutr..

[25] Hang D., Kvaerner A.S., Ma W., Hu Y., Tabung F.K., Nan H., Hu Z., Shen H., Mucci L.A., Chan A.T. (2019). Coffee consumption and plasma biomarkers of metabolic and inflammatory pathways in US health professionals. Am. J. Clin. Nutr..

[26] Kempf K., Herder C., Erlund I., Kolb H., Martin S., Carstensen M., Koenig W., Sundvall J., Bidel S., Kuha S. (2010). Effects of coffee consumption on subclinical inflammation and other risk factors for type 2 diabetes: A clinical trial. Am. J. Clin. Nutr..

[27] Loftfield E., Shiels M.S., Graubard B.I., Katki H.A., Chaturvedi A.K., Trabert B., Pinto L.A., Kemp T.J., Shebl F.M., Mayne S.T. (2015). Associations of coffee drinking with systemic immune and inflammatory markers. Cancer Epidemiol. Biomark. Prev..

[28] Nieman D.C., Goodman C.L., Capps C.R., Shue Z.L., Arnot R. (2018). Influence of 2-weeks ingestion of high chlorogenic acid coffee on mood state, performance, and postexercise inflammation and oxidative stress: A randomized, placebo-controlled trial. Int. J. Sport Nutr. Exerc. Metab..

[29] Martinez-Lopez S., Sarria B., Mateos R., Bravo-Clemente L. (2019). Moderate consumption of a soluble green/roasted coffee rich in caffeoylquinic acids reduces cardiovascular risk markers: Results from a randomized, cross-over, controlled trial in healthy and hypercholesterolemic subjects. Eur. J. Nutr..

[30] Correa T.A., Rogero M.M., Mioto B.M., Tarasoutchi D., Tuda V.L., Cesar L.A., Torres E.A. (2013). Paper-filtered coffee increases cholesterol and inflammation biomarkers independent of roasting degree: A clinical trial. Nutrition.

[31] Kempf K., Kolb H., Gartner B., Bytof G., Stiebitz H., Lantz I., Lang R., Hofmann T., Martin S. (2015). Cardiometabolic effects of two coffee blends differing in content for major constituents in overweight adults: A randomized controlled trial. Eur. J. Nutr..

[32] Lamichhane D., Collins C., Constantinescu F., Walitt B., Pettinger M., Parks C., Howard B.V. (2019). Coffee and tea consumption in relation to risk of rheumatoid arthritis in the women’s health initiative observational cohort. J. Clin. Rheumatol..

[33] Del Rio D., Rodriguez-Mateos A., Spencer J.P., Tognolini M., Borges G., Crozier A. (2013). Dietary (poly)phenolics in human health: Structures, bioavailability, and evidence of protective effects against chronic diseases. Antioxid. Redox. Signal..

[34] Fraga C.G., Croft K.D., Kennedy D.O., Tomas-Barberan F.A. (2019). The effects of polyphenols and other bioactives on human health. Food Funct..

[35] Burkholder-Cooley N., Rajaram S., Haddad E., Fraser G.E., Jaceldo-Siegl K. (2016). Comparison of polyphenol intakes according to distinct dietary patterns and food sources in the Adventist Health Study-2 cohort. Br. J. Nutr..

[36] Grosso G., Stepaniak U., Topor-Madry R., Szafraniec K., Pajak A. (2014). Estimated dietary intake and major food sources of polyphenols in the Polish arm of the HAPIEE study. Nutrition.

[37] Taguchi C., Fukushima Y., Kishimoto Y., Suzuki-Sugihara N., Saita E., Takahashi Y., Kondo K. (2015). estimated dietary polyphenol intake and major food and beverage sources among elderly Japanese. Nutrients.

[38] Zamora-Ros R., Rothwell J.A., Scalbert A., Knaze V., Romieu I., Slimani N., Fagherazzi G., Perquier F., Touillaud M., Molina-Montes E. (2013). Dietary intakes and food sources of phenolic acids in the European Prospective Investigation into Cancer and Nutrition (EPIC) study. Br. J. Nutr..

[39] Zamora-Ros R., Knaze V., Rothwell J.A., Hemon B., Moskal A., Overvad K., Tjonneland A., Kyro C., Fagherazzi G., Boutron-Ruault M.C. (2016). Dietary polyphenol intake in Europe: The European Prospective Investigation into Cancer and Nutrition (EPIC) study. Eur. J. Nutr..

[40] Qin S., Hou D.X. (2016). Multiple regulations of Keap1/Nrf2 system by dietary phytochemicals. Mol. Nutr. Food Res..

[41] Otsuki A., Yamamoto M. (2020). Cis-element architecture of Nrf2-sMaf heterodimer binding sites and its relation to diseases. Arch. Pharm. Res..

[42] Tebay L.E., Robertson H., Durant S.T., Vitale S.R., Penning T.M., Dinkova-Kostova A.T., Hayes J.D. (2015). Mechanisms of activation of the transcription factor Nrf2 by redox stressors, nutrient cues, and energy status and the pathways through which it attenuates degenerative disease. Free Radic. Biol. Med..

[43] Merry T.L., Ristow M. (2016). Nuclear factor erythroid-derived 2-like 2 (NFE2L2, Nrf2) mediates exercise-induced mitochondrial biogenesis and the anti-oxidant response in mice. J. Physiol..

[44] Coleman V., Sa-Nguanmoo P., Koenig J., Schulz T.J., Grune T., Klaus S., Kipp A.P., Ost M. (2018). Partial involvement of Nrf2 in skeletal muscle mitohormesis as an adaptive response to mitochondrial uncoupling. Sci. Rep..

[45] Tsushima M., Liu J., Hirao W., Yamazaki H., Tomita H., Itoh K. (2020). Emerging evidence for crosstalk between Nrf2 and mitochondria in physiological homeostasis and in heart disease. Arch. Pharm. Res..

[46] Unoki T., Akiyama M., Kumagai Y. (2020). Nrf2 activation and its coordination with the protective defense systems in response to electrophilic stress. Int. J. Mol. Sci..

[47] Baird L., Yamamoto M. (2020). The molecular mechanisms regulating the KEAP1-NRF2 pathway. Mol. Cell. Biol..

[48] Shin W.H., Park J.H., Chung K.C. (2020). The central regulator p62 between ubiquitin proteasome system and autophagy and its role in the mitophagy and Parkinson’s disease. BMB Rep..

[49] Cho H.Y., Miller-DeGraff L., Blankenship-Paris T., Wang X., Bell D.A., Lih F., Deterding L., Panduri V., Morgan D.L., Yamamoto M. (2019). Sulforaphane enriched transcriptome of lung mitochondrial energy metabolism and provided pulmonary injury protection via Nrf2 in mice. Toxicol. Appl. Pharmacol..

[50] Boyanapalli S.S., Paredes-Gonzalez X., Fuentes F., Zhang C., Guo Y., Pung D., Saw C.L., Kong A.N. (2014). Nrf2 knockout attenuates the anti-inflammatory effects of phenethyl isothiocyanate and curcumin. Chem. Res. Toxicol..

[51] Ungvari Z., Bagi Z., Feher A., Recchia F.A., Sonntag W.E., Pearson K., De Cabo R., Csiszar A. (2010). Resveratrol confers endothelial protection via activation of the antioxidant transcription factor Nrf2. Am. J. Physiol. Heart Circ. Physiol..

[52] Dludla P.V., Muller C.J., Joubert E., Louw J., Essop M.F., Gabuza K.B., Ghoor S., Huisamen B., Johnson R. (2017). Aspalathin protects the heart against hyperglycemia-induced oxidative damage by up-regulating Nrf2 expression. Molecules.

[53] Furue M., Uchi H., Mitoma C., Hashimoto-Hachiya A., Chiba T., Ito T., Nakahara T., Tsuji G. (2017). Antioxidants for healthy skin: The emerging role of aryl hydrocarbon receptors and nuclear factor-erythroid 2-related factor-2. Nutrients.

[54] Neavin D.R., Liu D., Ray B., Weinshilboum R.M. (2018). The role of the Aryl Hydrocarbon Receptor (AHR) in immune and inflammatory diseases. Int. J. Mol. Sci..

[55] Rothhammer V., Quintana F.J. (2019). The aryl hydrocarbon receptor: An environmental sensor integrating immune responses in health and disease. Nat. Rev. Immunol..

[56] Boettler U., Sommerfeld K., Volz N., Pahlke G., Teller N., Somoza V., Lang R., Hofmann T., Marko D. (2011). Coffee constituents as modulators of Nrf2 nuclear translocation and ARE (EpRE)-dependent gene expression. J. Nutr. Biochem..

[57] Cavin C., Marin-Kuan M., Langouet S., Bezencon C., Guignard G., Verguet C., Piguet D., Holzhauser D., Cornaz R., Schilter B. (2008). Induction of Nrf2-mediated cellular defenses and alteration of phase I activities as mechanisms of chemoprotective effects of coffee in the liver. Food Chem. Toxicol..

[58] Kalthoff S., Ehmer U., Freiberg N., Manns M.P., Strassburg C.P. (2010). Coffee induces expression of glucuronosyltransferases by the aryl hydrocarbon receptor and Nrf2 in liver and stomach. Gastroenterology.

[59] Salomone F., Li V.G., Vitaglione P., Morisco F., Fogliano V., Zappala A., Palmigiano A., Garozzo D., Caporaso N., D’Argenio G. (2014). Coffee enhances the expression of chaperones and antioxidant proteins in rats with nonalcoholic fatty liver disease. Transl. Res..

[60] Shi A., Shi H., Wang Y., Liu X., Cheng Y., Li H., Zhao H., Wang S., Dong L. (2018). Activation of Nrf2 pathway and inhibition of NLRP3 inflammasome activation contribute to the protective effect of chlorogenic acid on acute liver injury. Int. Immunopharmacol..

[61] Vicente S.J., Ishimoto E.Y., Torres E.A. (2014). Coffee modulates transcription factor Nrf2 and highly increases the activity of antioxidant enzymes in rats. J. Agric. Food Chem..

[62] Volz N., Boettler U., Winkler S., Teller N., Schwarz C., Bakuradze T., Eisenbrand G., Haupt L., Griffiths L.R., Stiebitz H. (2012). Effect of coffee combining green coffee bean constituents with typical roasting products on the Nrf2/ARE pathway in vitro and in vivo. J. Agric. Food Chem..

[63] Jung K.A., Kwak M.K. (2010). The Nrf2 system as a potential target for the development of indirect antioxidants. Molecules.

[64] Higgins L.G., Cavin C., Itoh K., Yamamoto M., Hayes J.D. (2008). Induction of cancer chemopreventive enzymes by coffee is mediated by transcription factor Nrf2. Evidence that the coffee-specific diterpenes cafestol and kahweol confer protection against acrolein. Toxicol. Appl. Pharmacol..

[65] Balstad T.R., Carlsen H., Myhrstad M.C., Kolberg M., Reiersen H., Gilen L., Ebihara K., Paur I., Blomhoff R. (2011). Coffee, broccoli and spices are strong inducers of electrophile response element-dependent transcription in vitro and in vivo—studies in electrophile response element transgenic mice. Mol. Nutr. Food Res..

[66] Boettler U., Volz N., Pahlke G., Teller N., Kotyczka C., Somoza V., Stiebitz H., Bytof G., Lantz I., Lang R. (2011). Coffees rich in chlorogenic acid or N-methylpyridinium induce chemopreventive phase II-enzymes via the Nrf2/ARE pathway in vitro and in vivo. Mol. Nutr. Food Res..

[67] Fratantonio D., Speciale A., Canali R., Natarelli L., Ferrari D., Saija A., Virgili F., Cimino F. (2017). Low nanomolar caffeic acid attenuates high glucose-induced endothelial dysfunction in primary human umbilical-vein endothelial cells by affecting NF-kappaB and Nrf2 pathways. Biofactors.

[68] Priftis A., Mitsiou D., Halabalaki M., Ntasi G., Stagos D., Skaltsounis L.A., Kouretas D. (2018). Roasting has a distinct effect on the antimutagenic activity of coffee varieties. Mutat. Res. Genet. Toxicol. Environ. Mutagen..

[69] Ren Y., Wang C., Xu J., Wang S. (2019). Cafestol and Kahweol: A Review on their bioactivities and pharmacological properties. Int. J. Mol. Sci..

[70] Paur I., Balstad T.R., Blomhoff R. (2010). Degree of roasting is the main determinant of the effects of coffee on NF-kappaB and EpRE. Free Radic. Biol. Med..

[71] Sauer T., Raithel M., Kressel J., Munch G., Pischetsrieder M. (2013). Activation of the transcription factor Nrf2 in macrophages, Caco-2 cells and intact human gut tissue by Maillard reaction products and coffee. Amino Acids.

[72] Murakami A. (2014). Dose-dependent functionality and toxicity of green tea polyphenols in experimental rodents. Arch. Biochem. Biophys..

[73] Karadas O., Mese G., Ozcivici E. (2019). Cytotoxic tolerance of healthy and cancerous bone cells to anti-microbial phenolic compounds depend on culture conditions. Appl. Biochem. Biotechnol..

[74] Wu H., Chen L., Zhu F., Han X., Sun L., Chen K. (2019). The cytotoxicity effect of resveratrol: Cell cycle arrest and induced apoptosis of breast cancer 4T1 Cells. Toxins (Basel).

[75] EFSA ANS Panel (2018). Scientific opinion on the safety of green tea catechins. EFSA J..

[76] Calabrese E.J. (2010). Hormesis is central to toxicology, pharmacology and risk assessment. Hum. Exp. Toxicol..

[77] Leri M., Scuto M., Ontario M.L., Calabrese V., Calabrese E.J., Bucciantini M., Stefani M. (2020). Healthy effects of plant polyphenols: Molecular mechanisms. Int. J. Mol. Sci..

[78] Miller V.J., Villamena F.A., Volek J.S. (2018). Nutritional ketosis and mitohormesis: Potential implications for mitochondrial function and human health. J. Nutr. Metab..

[79] Kolb H., Eizirik D.L. (2011). Resistance to type 2 diabetes mellitus: A matter of hormesis?. Nat. Rev. Endocrinol..

[80] Calabrese E.J., Mattson M.P. (2017). How does hormesis impact biology, toxicology, and medicine?. NPJ Aging Mech. Dis..

[81] Bakuradze T., Lang R., Hofmann T., Eisenbrand G., Schipp D., Galan J., Richling E. (2015). Consumption of a dark roast coffee decreases the level of spontaneous DNA strand breaks: A randomized controlled trial. Eur. J. Nutr..

[82] Schipp D., Tulinska J., Sustrova M., Liskova A., Spustova V., Lehotska M.M., Krivosikova Z., Rausova K., Collins A., Vebraite V. (2019). Consumption of a dark roast coffee blend reduces DNA damage in humans: Results from a 4-week randomised controlled study. Eur. J. Nutr..

[83] Pahlke G., Attapah E., Aichinger G., Ahlberg K., Hochkogler C., Schipp D., Somoza V., Marko D. (2019). Dark coffee consumption protects human blood cells from spontaneous DNA damage. J. Funct. Foods.

[84] EFSA Panel on NDA (2020). Scientifc opinion on Coffee C21 and protection of DNA from strand breaks. EFSA J..

[85] Valverde M., Rojas E. (2009). Environmental and occupational biomonitoring using the Comet assay. Mutat. Res..

[86] Jaquet M., Rochat I., Moulin J., Cavin C., Bibiloni R. (2009). Impact of coffee consumption on the gut microbiota: A human volunteer study. Int. J. Food Microbiol..

[87] Cowan T.E., Palmnas M.S., Yang J., Bomhof M.R., Ardell K.L., Reimer R.A., Vogel H.J., Shearer J. (2014). Chronic coffee consumption in the diet-induced obese rat: Impact on gut microbiota and serum metabolomics. J. Nutr. Biochem..

[88] Nakayama T., Oishi K. (2013). Influence of coffee (*Coffea arabica*) and galacto-oligosaccharide consumption on intestinal microbiota and the host responses. FEMS Microbiol. Lett..

[89] Gniechwitz D., Brueckel B., Reichardt N., Blaut M., Steinhart H., Bunzel M. (2007). Coffee dietary fiber contents and structural characteristics as influenced by coffee type and technological and brewing procedures. J. Agric. Food Chem..

[90] Williamson G. (2017). The role of polyphenols in modern nutrition. Nutr. Bull..

[91] Perez-Burillo S., Rajakaruna S., Pastoriza S., Paliy O., Rufian-Henares J.A. (2020). Bioactivity of food melanoidins is mediated by gut microbiota. Food Chem..

[92] Nishitsuji K., Watanabe S., Xiao J., Nagatomo R., Ogawa H., Tsunematsu T., Umemoto H., Morimoto Y., Akatsu H., Inoue K. (2018). Effect of coffee or coffee components on gut microbiome and short-chain fatty acids in a mouse model of metabolic syndrome. Sci. Rep..

[93] Saeedi B.J., Liu K.H., Owens J.A., Hunter-Chang S., Camacho M.C., Eboka R.U., Chandrasekharan B., Baker N.F., Darby T.M., Robinson B.S. (2020). Gut-resident lactobacilli activate hepatic Nrf2 and protect against oxidative liver injury. Cell Metab..

[94] Fragiadakis G.K., Wastyk H.C., Robinson J.L., Sonnenburg E.D., Sonnenburg J.L., Gardner C.D. (2020). Long-term dietary intervention reveals resilience of the gut microbiota despite changes in diet and weight. Am. J. Clin. Nutr..

[95] Son T.G., Camandola S., Mattson M.P. (2008). Hormetic dietary phytochemicals. Neuromolecular Med..

[96] Martel J., Ojcius D.M., Ko Y.F., Ke P.Y., Wu C.Y., Peng H.H., Young J.D. (2019). Hormetic effects of phytochemicals on health and longevity. Trends Endocrinol. Metab..

[97] Yahfoufi N., Alsadi N., Jambi M., Matar C. (2018). The immunomodulatory and anti-inflammatory role of polyphenols. Nutrients.

[98] Liu K., Luo M., Wei S. (2019). The bioprotective effects of polyphenols on metabolic syndrome against oxidative stress: Evidences and perspectives. Oxid. Med. Cell Longev..

[99] Palliyaguru D.L., Chartoumpekis D.V., Wakabayashi N., Skoko J.J., Yagishita Y., Singh S.V., Kensler T.W. (2016). Withaferin A induces Nrf2-dependent protection against liver injury: Role of Keap1-independent mechanisms. Free Radic. Biol. Med..

[100] Hao Q., Wang M., Sun N.X., Zhu C., Lin Y.M., Li C., Liu F., Zhu W.W. (2020). Sulforaphane suppresses carcinogenesis of colorectal cancer through the ERK/Nrf2UDP glucuronosyltransferase 1A metabolic axis activation. Oncol. Rep..

[101] Zhao S.J., Liu H., Chen J., Qian D.F., Kong F.Q., Jie J., Yin G.Y., Li Q.Q., Fan J. (2020). Macrophage GIT1 contributes to bone regeneration by regulating inflammatory responses in an ERK/NRF2-dependent way. J. Bone Miner. Res..

[102] Lei L., Chai Y., Lin H., Chen C., Zhao M., Xiong W., Zhuang J., Fan X. (2020). Dihydroquercetin activates AMPK/Nrf2/HO-1 signaling in macrophages and attenuates inflammation in LPS-induced endotoxemic mice. Front. Pharmacol..

[103] Lu J., Huang Q., Zhang D., Lan T., Zhang Y., Tang X., Xu P., Zhao D., Cong D., Zhao D. (2020). The protective effect of DiDang Tang against AlCl3-Induced oxidative stress and apoptosis in PC12 cells through the activation of SIRT1-mediated Akt/Nrf2/HO-1 pathway. Front. Pharmacol..

